# Electrical impedance tomography: so close to touching the holy grail

**DOI:** 10.1186/cc13979

**Published:** 2014-07-08

**Authors:** Jean-Michel Constantin, Sebastien Perbet, Julie Delmas, Emmanuel Futier

**Affiliations:** 1 Department of Perioperative Medicine, University Hospital of Clermont-Ferrand, 1 place Lucie Aubrac, 63003 Clermont-Ferrand, France

## Abstract

Electrical impedance tomography is a new technology giving us lung imaging that may allow lung function to be monitored at the bedside. Several applications have been studied to guide mechanical ventilation at the bedside with electrical impedance tomography. Positive end-expiratory pressure trials guided by electrical impedance tomography are relevant in terms of recruited volume or homogeneity of the lung. Tidal impedance variation is a new parameter of electrical impedance tomography that may help physicians with ventilator settings in acute respiratory distress syndrome patients. This parameter is able to identify the onset of overdistention in the nondependent part and recruitment in the dependent part. Electrical impedance tomography presents a big step forward in mechanical ventilation.

## 

In a previous issue of *Critical Care*, Blankman and coworkers provide new results that may change the mechanical ventilation paradigm [1]. Over the past 25 years, the use of computed tomography (CT) for clinical evaluation of thoracic diseases has rapidly gained in popularity and CT has become firmly established as an important research and diagnostic modality [2]. CT yields cross-sectional images of the lung, allowing virtually all intrathoracic pathological conditions to be evaluated. In particular, CT has played an important role in improving our knowledge of the pathophysiology of acute respiratory distress syndrome (ARDS) and in determining the morphological and functional consequences of different manoeuvres commonly used in the therapeutic management of this syndrome [3]. Gattinoni and colleagues’ [3] and Rouby and colleagues’ [2] trials of CT have definitively changed our meaning of ARDS, but this remains research; CT still needs patient transfer to the department of radiology, CT still needs radiation, and CT is still static imaging. In fact, physicians are still blind at the bedside.

Electrical impedance tomography (EIT) is a new technology giving us bedside lung imaging that may allow lung function to be monitored at the bedside. Several applications have been studied to guide mechanical ventilation at the bedside with EIT. Positive end-expiratory pressure (PEEP) trials guided by EIT are relevant in terms of recruited volume or homogeneity of the lung [4]. Setting the ventilator to avoid alveolar derecruitment, loss of aeration, is crucial both in anaesthesia and in the ICU [5]. In ICU patients with ARDS, however, looking for alveolar recruitment is insufficient. In ARDS patients, even the normally aerated lung is not a healthy lung and overdistention may occur [6]. Overdistention, with or without hyperinflation, is defined as an excess of alveolar gas compared with lung tissue. Very probably, 90 % lung aeration (corresponding to CT attenuations of -900 Hounsfield Units or less) is the threshold that separates inflation from overinflation. If inflation of healthy lung regions during mechanical ventilation is safe (but useless), hyperinflation-induced overdistention of nondependent lung regions may be harmful and is probably a major issue in mechanical ventilation, responsible for ventilator-induced lung injury [2].

If surrogates of recruited volume have been extensively described in the last decade (with EIT, bedside functional residual capacity, dynamic compliance, lung echo, and so forth), the visualisation of overdistention remains CT’s prerogative. Lung overdistention is therefore a long-running story between myth and fact.

In the late 1990s, pressure–volume curve analysis was identified as a good surrogate of the recruitment–overdistention relationship [7]. Unfortunately, results were disappointing. Indeed, recruitment is a continuous phenomenon that may occur after the upper inflection point, and overdistention may appear before this point [8]. More recently, studies with lung ultrasound showed the possibility to quantify the recruitment, but with a subjective analysis and the inability to measure the overdistention [9].

Blankman and coworkers used a new EIT parameter, tidal impedance variation, to find the ‘best’ PEEP, which is regarded as minimal lung collapse and minimal overdistention in order to prevent ventilator-induced lung injury [1]. During a decremental PEEP trial, they have shown that intratidal gas distribution is able to identify the onset of overdistention in the nondependent part and recruitment in the dependent part. Looking for lung overdistention at the bedside, in addition to recruited volume, looks like the holy grail for physicians dealing with ARDS over the years. Because alveolar recruitment is a continuous phenomenon during lung inflation, and because keeping the plateau pressure below 30 cmH_2_O with a reduced tidal volume does not protect all patients from overdistention [10], taking into account overdistention during PEEP trials is a crucial issue. These preliminary results obtained in postcardiac surgery patients are therefore a big step forward in mechanical ventilation. Obviously this study has several limits. The most important, in our view, is that this study was performed with postcardiac surgery patients. ARDS after cardiac surgery is known to be really diffuse with a homogeneous loss of aeration. But even this limit is not a real issue. We shall have to perform the same study with different ICU patients. The proof of concept is done, and that is most important.

EIT is probably a revolution in mechanical ventilation. Even if EIT devices moved from research laboratories to ICUs, this revolution is ongoing [11]. The results of this study are one more proof of EIT’s ability to change the paradigm of mechanical ventilation, but obviously not the last. An increasing use in the clinical setting can be forecast in areas other than lung aeration/lung collapse. As the perfusion-related changes in thoracic impedance are about one order of magnitude smaller than the changes induced by ventilation, it is much more difficult to extract information on stroke volume, cardiac output, or lung perfusion. Notwithstanding, separation of cardiac-related and ventilation-related EIT signals is not impossible, as shown recently [12]. Ventilation–perfusion monitoring continuously at the bedside for ICU patients during PEEP trials, prone positioning, or recruitment manoeuvres may be the next step – and yes, this will be the holy grail!

## Abbreviations

ARDS: Acute respiratory distress syndrome; CT: Computed tomography; EIT: Electrical impedance tomography; PEEP: Positive end-expiratory pressure.

## Competing interests

J-MC and EF have received fees for lectures from GE and Dragger. The remaining authors declare that they have no competing interests.
